# CDK4/6 Inhibitors in Pancreatobiliary Cancers: Opportunities and Challenges

**DOI:** 10.3390/cancers15030968

**Published:** 2023-02-03

**Authors:** Tatjana Arsenijevic, Katia Coulonval, Eric Raspé, Anne Demols, Pierre P. Roger, Jean-Luc Van Laethem

**Affiliations:** 1 Laboratory of Experimental Gastroenterology, Université Libre de Bruxelles, Route de Lennik 808, 1070 Brussels, Belgium; 2 Department of Gastroenterology, Hepatology and Digestive Oncology, HUB Bordet Erasme Hospital, Université Libre de Bruxelles, Route de Lennik 808, 1070 Brussels, Belgium; 3 Institute of Interdisciplinary Research (Iribhm), ULB-Cancer Research Center (U-crc), Université Libre de Bruxelles, Campus Erasme, Route de Lennik 808, 1070 Brussels, Belgium

**Keywords:** pancreatic ductal adenocarcinoma, cholangiocarcinoma, CDK4/6 inhibitors

## Abstract

**Simple Summary:**

Pancreatobiliary cancers are a group of malignancies affecting the pancreas and biliary tract and are among the cancers with the lowest survival rate. Current first-line treatments only offer a modest increase in overall survival, and there is an urgent need to develop new therapeutic strategies. Emerging evidence suggests cyclin-dependent kinase 4/6 (CDK4/6) inhibitors as an attractive therapeutic strategy for solid cancers, and here, we summarize the current knowledge and exploration of their therapeutic potential in the most common pancreatobilliary cancers, cholangiocarcinoma and pancreatic ductal adenocarcinoma.

**Abstract:**

Existing treatment strategies for pancreatobiliary malignancies are limited. Nowadays, surgery is the only path to cure these types of cancer, but only a small number of patients present with resectable tumors at the time of diagnosis. The notoriously poor prognosis, lack of diverse treatment options associated with pancreaticobiliary cancers, and their resistance to current therapies reflect the urge for the development of novel therapeutic targets. Cyclin-dependent kinase 4/6 (CDK4/6) inhibitors have emerged as an attractive therapeutic strategy in a number of cancers since their approval for treatment in patients with ER+/HER- breast cancer in combination with antiestrogens. In this article, we discuss the therapeutic potential of CDK4/6 inhibitors in pancreatobiliary cancers, notably cholangiocarcinoma and pancreatic ductal adenocarcinoma.

## 1. Introduction

### 1.1. Cell Cycle Control

**Figure 1:**
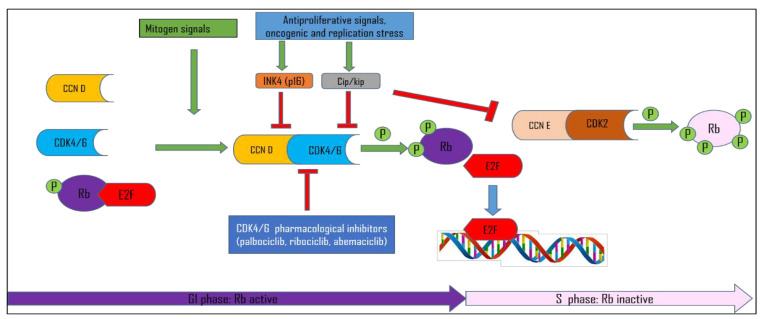
RB pathway and G1/S cell cycle control. Cyclin (CCN); Cyclin-dependent kinase (CDK); Retinoblastoma protein (RB); E2F transcription factor (E2F).

The cell cycle is a highly organized four-stage process in which the cell increases its size (G1 stage), copies its DNA (S stage), prepares to divide (G2 stage), and divides (M stage). Cell cycle progression is controlled by the expression and activation of various cyclin-dependent kinases (CDKs). CDKs are serine-threonine kinases that, to be activated, require, besides post-translational modifications (phosphorylation), the association with their regulatory proteins, called cyclins [1,2]. CDK4/6 regulate the G1 phase of the cell cycle when associated with their regulatory subunits cyclins D (CCND1-3) [1,3]. CDK4 and CDK6 are expressed at constant levels throughout the cell cycle. By contrast, D-cyclins are labile proteins, transcriptionally induced upon the stimulation of cells with growth factors. Upon proper mitogenic stimulation, cyclins D accumulate and form a complex with CDK4/6 activating them to phosphorylate the retinoblastoma protein RB and two other retinoblastoma family members, p107 and p130 [4,5] (Figure 1). However, CDK4 activation is much more complex, and besides the assembly of cyclin D–CDK4 complexes, it requires complex stabilization by binding to p21 or p27 proteins, complex nuclear import, and last but not least, the activating T-loop phosphorylation of CDK4 at Thr172 [4,6]. This event has been shown to be the central rate-limiting event in CDK4 activation and determines RB phosphorylation and cell cycle commitment in RB-proficient cells [7,8,9].

Initially, the phosphorylation of RB by cyclin D–CDK4/6 was thought to partially inactivate RB, leading to the release of E2F (E2F family of transcription factors) and up-regulation of E2F-transcriptional targets, including cyclin E. Cyclin E subsequently forms a complex with its kinase partner, CDK2, and completes full RB phosphorylation, creating a positive feedback loop in which RB is maintained in a phosphorylated state throughout the transition into S phase. This is called the restriction point (R), and any cell getting past the restriction point is committed to the S phase and ultimately, mitotic division [10,11]. More recent data speculate that D-type cyclin–CDK complexes are not involved in the initial inactivation of RB and E2F-dependent transcription but rather create a state between the cell cycle exit (quiescence) and the cell cycle entry [12,13]. However, recent single-cell analyses confirmed that cyclin D–CDK4/6 activity is required for the hyperphosphorylation of RB throughout G1, whereas cyclin E/A–CDK is responsible for the maintenance of RB in a hyperphosphorylated state in the S phase [14]. Furthermore, it has been shown recently that both noncancer and cancer cells bifurcate into two subpopulations after anaphase, marked by increasing vs. low CDK2 activity and the hyper- vs. hypophosphorylation of RB, delineating one subpopulation that never “uncrosses” the restriction point and continues cycling and another subpopulation that exits mitosis into an uncommitted pre-restriction point state [15]. Besides binding to cyclins D, CDK4/6 activity during G1–S transition is regulated by two CDK inhibitor families: Cip/Kip (p21cip1, p27kip1, and p57kip2) and Ink4 (p16Ink4a, p15Ink4b, p18Ink4c, and p19Ink4d) [16] (Figure 1). The Cip/Kip family broadly inhibits the cyclin D–CDK4/6 complex activity and the activity of CDK2-containing complexes, [17,18]. Nonetheless, it has been shown that cip/kip family members can serve as assembly factors for cyclin D–CDK4/6 [2,16,19]. The explanation for this controversy lies in the fact that post-translational modifications of p21 and p27 proteins dictate their inhibitor/non-inhibitor role [20]. Hereby, the phosphorylation of p21 and p27 was found to be critical for CDK4 Thr172-phosphorylation and activation [9,21,22,23,24].

Ink4 family proteins specifically bind and inactivate CDK4/6, thus inducing the inhibition of RB phosphorylation and blocking cell cycle progression [2,16,20,25]. The interaction among cyclin D/CDK, p16Ink4A, and RB/E2F constitutes a functional unit known as the “RB pathway”. As cancer is a disease of the deregulated cell cycle, the RB pathway is affected in many tumors [26,27,28]. The RB pathway can be deregulated through various genetic alterations, such as the amplification of CDK4/CDK6 and D-type cyclin genes, deletion or silencing of the *CDKN2A/B* gene encoding the Ink4 inhibitors p16 and p15, or the loss of or mutations in RB itself [26,29]. However, as most human tumors retain intact RB, the cell cycle arrest can be best achieved by the direct inhibition of CDK4/CDK6. This provided a strong rationale for designing and testing synthetic inhibitors of CDK4/6 as potential anticancer drugs.

### 1.2. CDK Inhibitors

Over the past three decades, numerous compounds targeting CDK activity have been developed and evaluated in preclinical and clinical trials [30]. The first-generation molecules, including flavopiridol and roscovitine, were potent inhibitors of numerous CDKs, so-called pan-CDK inhibitors, which resulted in their poor selectivity and high toxicity and the majority of them were never approved for clinical use [31,32,33,34].

Second-generation pan-CDK inhibitors were developed with a much higher affinity for specific CDKs compared to first-generation inhibitors, but unfortunately, they have shown to have limited clinical activity [35,36]. Much effort is devoted to the development of new specific inhibitors of CDK7 and CDK2 [37,38].

**Figure 2:**
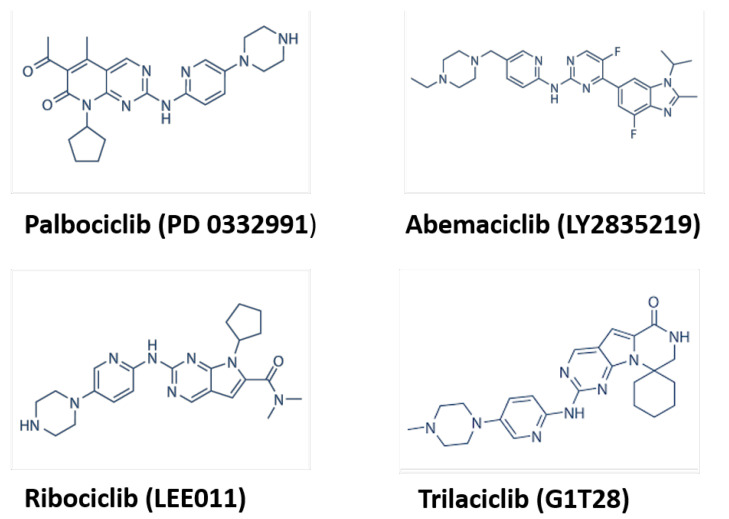
Chemical structures of CDK4/6 inhibitors (source: selleckchem.com (accessed on 16 January 2023)).

The first group of CDK-selective compounds to enter the clinics was the third-generation, CDK4/6 specific inhibitors, called palbociclib (PD0332991), ribociclib (LEE-011), and abemaciclib (LY2835219) [6]. In 2021, a 4th CDK4/6 inhibitor, trilaciclib, or G1T28, received FDA approval to reduce the incidence of chemotherapy-induced myelosuppression in patients before topotecan-containing or platinum and etoposide-containing chemotherapy for extensive-stage small cell lung cancer [39,40] (Figure 2). Palbociclib, ribociclib, and abemaciclib are orally active, highly selective inhibitors of the cyclin D-dependent kinases CDK4/6 with the ability to block RB phosphorylation in the nanomolar range. This prevents RB phosphorylation at serine 780 and 795, preventing the cell from passing the restriction point R and inducing G1 arrest [6]. They have similar tumor-suppressing functions as Ink4 family members [41]. All three CDK4/6 inhibitors bind and inhibit CDK4/6 within a nanomolar range; however, abemaciclib targets other CDKs as well [42,43,44,45]. Owing to its unprecedented efficacy based on progression-free survival, palbociclib was granted accelerated approval by the FDA in 2015 for the treatment of estrogen receptor (ER)-positive, human epidermal growth factor receptor 2 (HER2)-negative advanced breast cancer as initial endocrine-based therapy in postmenopausal women [46]. Besides breast cancer, CDK4/6 inhibitors have been shown as a promising therapy for several other cancers, including glioma [47], multiple myeloma [48], and liposarcoma [49], and are currently being evaluated for a variety of other cancers [20,50]. The major adverse reactions of CDK4/6 inhibitors are leukopenia and neutropenia, mainly caused by palbociclib and ribociclib, and for this reason, they are dosed with three weeks of treatment and one week of treatment discontinuation to recover neutrophil counts [46]. In contrast with those of palbociclib and ribociclib, the predominant toxicities of abemaciclib are diarrhea and fatigue with milder neutropenia. This toxicity profile allows for the continuous dosing of this agent [51]. In contrast to chemotherapy, CDK4/6 inhibitor-associated neutropenia is rapidly reversible, as CDK4/6 inhibitors induce cell-cycle arrest by decreasing the proliferation of hematopoietic stem cells, with resumed proliferation following a CDK4/6 dose reduction or interruption [52]. All CDK4/6 inhibitors can also cause gastrointestinal adverse effects, including diarrhea, nausea, and vomiting, which could be minimized by careful monitoring [53].

### 1.3. Pancreatobiliary Cancers

Pancreatobiliary cancers are a group of malignancies affecting the pancreas and biliary tract and are among the cancers with the lowest survival rate.

#### 1.3.1. Cholangiocarcinoma

Cholangiocarcinoma (CCA) constitutes a heterogeneous group of malignancies that can emerge at any point in the biliary tree [54]. It is a rare and aggressive malignancy, characterized by early lymph node involvement and distant metastasis, with 5-year survival rates between 5% and 15% depending on the type and stage [55]. CCA can be intrahepatic (iCCA) and extrahepatic (eCAA). This anatomical heterogeneity is translated in terms of biomolecular heterogeneity; for example, iCCA has a higher frequency of FGFR2 (fibroblast growth factor receptor 2) fusions or IDH1 or IDH2 (isocitrate dehydrogenase 1 or 2) mutations than eCCA [56,57,58,59].

**Figure 3:**
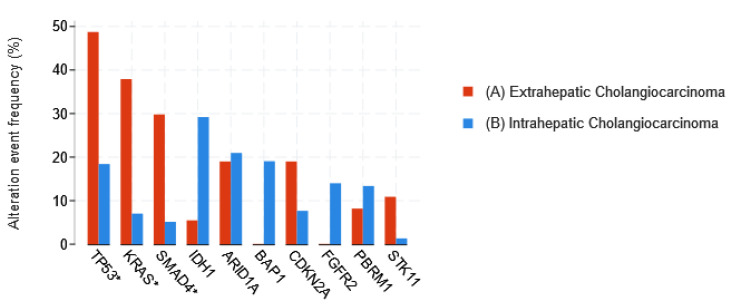
Most frequent genomic alterations in eCCA and iCCA, (*) genes with the highest alteration frequency [59] extracted from (http://cbioportal.org (accessed on 16 January 2023)) [70,71].

**Figure 4:**
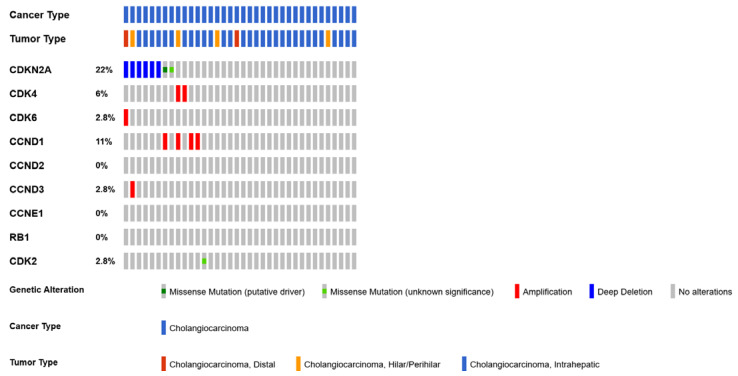
OncoPrint of the RB pathway alterations in CCA, showing mutations and copy number alterations (The Cancer Genome Atlas Program (TCGA)).

CCA has a notoriously poor prognosis. Radical surgical resection with a microscopic tumor-free resection margin (R0) remains the mainstay of potentially curative treatment for all three disease subtypes [60]. However, even after surgery, the 5-year overall survival remains poor. Evidence for adjuvant treatment (chemotherapy with or without radiotherapy) was weak [61], but the standard of care is now capecitabine, [62,63,64]. For patients with the unresectable or metastatic disease, the combination of gemcitabine–cisplatin+durvalumab is recommended as a first line regimen, with FOLFOX (folinic acid, fluorouracil, and oxaliplatin) as the second line [65,66,67]. The RAS/RAF/MEK and the mitogen-activated protein kinase/extracellular signal-regulated kinase (MAPK/ERK) pathway is defective in a large number of epithelial tumors including CCAs. Mutations in the proto-oncogene *KRAS* (Kirsten rat sarcoma virus) are frequently reported in CCA, as well as alterations of the *p53* gene and *CDKN2A* (cyclin-dependent kinase inhibitor 2A gene), being more prevalent in eCCA than in iCCA [56,59] (Figure 3). *CDKN2A* loss is reported in a significant proportion of iCCAs [68] (Figure 4). Furthermore, the Ink4a–ARF locus, which encodes two members of the Ink4 family (p16 and p14), is frequently inactivated in CCA [69]. In addition to KRAS, alterations of the TP53, CDKN2A/B, and the MAPK/ERK pathway have been significantly correlated with worse survival in CCA, with no OS difference with respect to the tumor location among iCCA, eCCA, and gall bladder [56,68].

#### 1.3.2. Pancreatic Ductal Adenocarcinoma

Pancreatic cancer, with pancreatic ductal adenocarcinoma (PDAC) as the most common type, remains one of the most aggressive and lethal malignancies, with a five-year survival of 9% [72]. While surgical resection is considered the only potentially curative treatment for PDAC, the recent developments in medical therapies, including active chemotherapy and radiotherapy, suggest that the combination of both, surgery and medical therapies, offers better outcomes [73,74,75]. Only one-half of patients with PDAC have non-metastatic disease at diagnosis, for whom a project of surgery could be considered [76,77]. Nowadays, two main chemotherapeutic regimens have proven their efficacy in PDAC management: FOLFIRINOX (irinotecan, oxaliplatin and leucovirin-modulated fluorouracil) and Nab-paclitaxel/gemcitabine in metastatic disease, and the first one is used in an adjuvant setting after surgery [78,79,80]. These regimens are now increasingly used in a neoadjuvant setting to downstage PDAC that is not upfront curatively resectable for surgical excision [81]. Despite recent improvements in the therapy of PDAC, the prognosis remains poor. To date, neither personalized medicine nor immunotherapy, proven successful in a variety of solid cancer therapies, have delivered major positive results in the treatment of PDAC.

**Figure 5:**
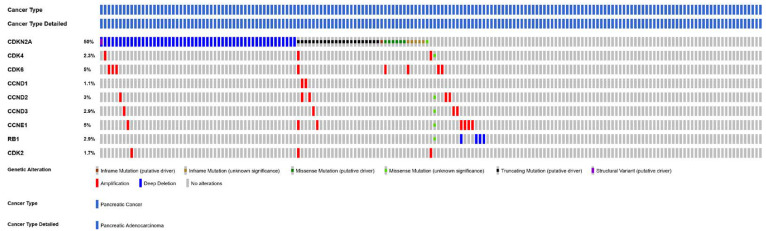
Oncoprint of the RB pathway genomic alterations in PDAC, showing mutations and copy number alterations (TCGA).

The molecular analyses of PDAC have revealed that four driver genes—*KRAS*, *CDKN2A, p53*, and *SMAD4* (SMAD family member 4)—are mutated in more than 50% of cases, with *KRAS* being an early mutational event found in more than 95% of invasive ductal adenocarcinomas [82,83,84]. Multiple genetic aberrations occurring in PDAC converge in the deregulation of the cyclin D-dependent kinases CDK4 and CDK6, which drive the G1–S phase transition of the cell cycle through inactivation of the RB pathway [85] (Figure 5). Mutant KRAS signaling leads to the induction of D-type cyclins, which enhances the kinase activities of CDK4 and CDK6 [86]. PDAC might have the highest probability of initial sensitivity to CDK4/6 inhibitors due to the frequent alteration of the CDKN2A locus through mutations, deletions, or epigenetic silencing, as well as an intact RB locus [87,88].

**Figure 6:**
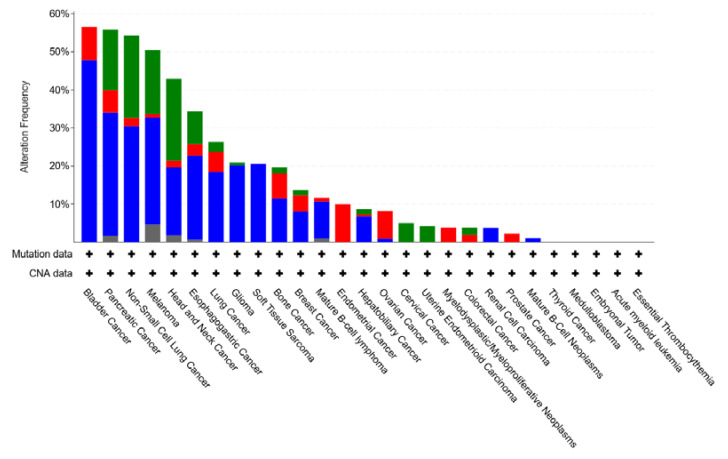
CDKN2A gene alterations in human cancers (TCGA pancancer data).

The TCGA pancancer study also teaches us that the proportion of tumors with mutated *CDKN2A* with respect to deletions is among the highest in pancreatic cancer (together with bladder cancer, non-small cell lung cancer, melanoma) (Figure 6).

## 2. CDK4/6 Inhibitors in Preclinical Models of CCA and PDAC

### 2.1. CDK4/6 Inhibitor Monotherapy in Preclinical Models of CCA and PDAC

The effects of CDK4/6 inhibitor monotherapy on CCA cell lines were reported in a limited number of studies with conflicting results.

Sitthithumcharee et al. showed that in 11 of 15 CCA cell lines tested, exposure of cells to palbociclib leads to cell cycle arrest and senescence [89]. It has been further shown that those cells sensitive to palbociclib express RB protein, and that the loss of RB conferred palbociclib resistance. The effectiveness of CDK4/6 inhibition for CCA was confirmed in 3D culture, xenograft, and patient-derived xenograft models (PDTX). Furthermore, the sensitivity of CCA to CDK4/6 inhibition has been associated with the activated KRAS signature [89]. However, in another study, no inhibitory effect of palbociclib was observed on the proliferation of several CCA cell lines [90]. This discrepancy might be explained by different methodologies of measurements of cell sensitivity to palbociclib and different drug exposure times. Sittithumcharee et al. treated cells for 5 days with increasing doses of palbociclib and measured the growth rate inhibition (GR50) based on the imaging of DAPI-stained cells. They confirmed the sensitivity data through the quantification of the proportions of cells in the various phases of the cell cycle via FACS analysis and metabolic labeling of DNA synthesis with EdU. By contrast, Saqub et al. measured cell viability utilizing highly water-soluble tetrazolium salt after 24 h of treatment with palbociclib. This last technology is much less reliable to evaluate the cytostatic effect of the CDK4/6 inhibitors. Raspé et al. showed a strong reduction of BrdU labeling in several breast cancer cell lines upon treatment with these drugs but no change when the impact of the drugs were measured via a viability assay with tetrazolium salts [91].

In pancreatic cancer models, data have been variable, with several studies demonstrating mono-therapy activity of CDK4/6is [92,93,94], but inherent resistance was also described [93,95,96].

One of the first studies testing the effects of palbociclib on PDAC cell lines demonstrated that palbociclib has antiproliferative effects on tumoral cells by downregulating cell cycle genes, but that at the same time, upregulated genes implicated in extracellular matrix (ECM) remodeling and pancreatic cancer cell invasion and metastasis [97]. The authors have shown that anti-CDK4/6 therapy could induce epithelial-to-mesenchymal transition and enhance PDAC cell invasion in *SMAD4* wild-type cells by activating SMAD-dependent transforming growth factor β (TGF-β) signaling and proposed a combination therapy of palbociclib with the type-I TGF-β receptor (TβRI) kinase inhibitor as a potential novel therapeutic strategy in PDAC.

The short-term anti-proliferative properties of CDK4/6is have been demonstrated in patient-derived cell lines [94]. In PDTXs of PDAC, palbociclib was highly efficient at suppressing proliferation in 14 of the 15 explants. In the single resistant explant, the rare loss of RB was identified as the basis for resistance [93]. Another study showed that a high RB expression profile in PDAC cell lines can be used to determine the sensitivity of cells to palbociclib treatment [98].

### 2.2. The Effects of CDK4/6 Inhibitors in Monotherapy: Quiescence, Senescence, or Resistance?

In cells with a functional RB, CDK4/6 inhibitors will inhibit CDK4/6 activity, block RB phosphorylation, and reduce E2F1 release, which results in G1 phase arrest. Depending on different factors, this inhibition would lead the cells into a state of quiescence, senescence, or apoptosis. Quiescence is a cellular state in which a cell exits the cell cycle but retains the capacity to divide. CDK4/6is are mainly cytostatic in vitro (halting the cell proliferation of sensitive cells but not killing them). Various models have shown that this proliferation arrest might become irreversible after long-term treatment (senescence). Findings described above showed that in CCA and PDAC preclinical models, CDK4/6 inhibitors used as single agents can induce cell cycle arrest and mostly senescence and to a much lesser extent, programmed cell death (apoptosis). Even though the induction of senescence contributes to tumor eradication, a durable response to monotherapy of CDK4/6 inhibitors has been shown to be challenging to achieve. While CDK4/6 inhibition has proven effective in clinical use in a subset of breast cancers, most patients eventually progress on treatment due to the adaptation and acquired resistance to CDK4/6 inhibition. Similarly, PDAC PDTXs treated with palbociclib exhibited potent adaptive responses and over the course of 21 days, many tumors started to progress with treatment. These adaptive responses included a rapid increase in the cyclin D1 and cyclin E1 protein synthesis rate that was associated with preserved signaling through the KRAS pathway [95]. Interestingly, in CCA cell models, cyclin D1 gene expression was reported to be elevated in cells that did not respond strongly to CDK4/6 inhibition [89,99]. The RB loss, although a rare event in PDAC, was attributed as a cause of CDK4/6i resistance in some PDAC models [93]. The research interest switched to combination therapies with CDK4/6 inhibitors that could avoid the CDK4/6 inhibitor resistance.

### 2.3. Combination Therapies to Overcome CDK4/6 Inhibitor Resistance

CDK4/6 inhibitors have been shown to have a more pronounced impact on proliferation in CCA and PDAC models in combination with other chemical agents. mTOR (mammalian target of rapamycin) signaling is one of the most involved pathways in the maintenance of cell survival and inhibition of apoptosis in solid tumors. The synergistic inhibitory activity of palbociclib and an mTOR inhibitor on iCCA cell proliferation has been reported [99]. Mechanistically, palbociclib potentiated mTOR inhibitory activity, whereas the mTOR inhibitor prevented the upregulation of cyclin D1 induced by palbociclib treatment [99]. Similarly to that in CCA models, in PDAC models, palbociclib resistance could be overcome with mTOR inhibitors [95].

Likewise, the inactivation of focal adhesion kinase (FAK) and CDK4/6 induced by a combination of a FAK inhibitor, PND1186, and palbociclib also showed greater antiproliferative effects on CCA in vitro and in vivo [100].

*KRAS* mutations are found in over 90% of PDACs resulting in hyperactivation of the MEK/ERK pathway. A combination of CDK4/6 inhibitors and MEK inhibitors inhibited growth and increased apoptosis in PDAC cell lines and patient-derived models of pancreatic cancer [94,101]. Combinations with MEK inhibitors are more likely to prevent the appearance of resistance to CDK4/6is and generate a senescence response in tumor cells, partly because they oppose the increased levels of cyclins D that are most often observed in response to CDK4/6is (due to stabilization inside CDK4/6 complexes, but also to increased AP1 (activator protein 1) transcriptional activity [102,103,104,105,106]).

KRAS-driven PDAC formation and maintenance have been demonstrated to depend on active phosphatidylinositol 3-kinase (PI3K) signaling. Insulin-like growth factor 1 (IGF1) receptor inhibitor, which potently inhibits the PI3K effector protein kinase B (AKT), synergizes with palbociclib to suppress growth and induce senescence in p16INK4A-deficient pancreatic cancers [96].

Abemaciclib has been shown to cause G1 arrest and inhibit the cell cycle in PDAC cells, and this effect was pronounced when combined with HuR (human antigen R) and YAP1 (yes1 -associated transcriptional regulator) inhibitors that regulate cyclin D1 expression [107].

Several studies have shown that resistance to CDK4/6 inhibitors results from the induction of autophagy in several solid tumors. Autophagy serves to recycle cellular constituents by engulfing them into vesicles called autophagosomes, which eventually fuse with lysosomes to facilitate the degradation of the cellular constituents and generate energy for survival [108]. A recent study demonstrated that the combination of CDK4/6 and autophagy inhibitors can be utilized to effectively treat several solid tumors, including PDAC, with RB and cyclin E proposed as biomarkers of the response [109].

### 2.4. CDK4/6 Inhibitors in Combination with Chemotherapy

As CDK4/6 inhibitors arrest cells in the G1 phase, they were initially considered to be incompatible in combination with DNA-damaging and antimitotic therapeutics, which are often used as a standard of care for most cancer types. In agreement with this, several early studies using CCA and PDAC models documented that the co-administration of CDK4/6 inhibitors antagonized the therapeutic effects of various classes of chemotherapeutic compounds, such as taxanes and cisplatin [92,94]. However, some cooperative effects of chemotherapeutics with CDK4/6is have been reported [94,110]. Using a combination of 2D and 3D in vitro and in vivo models, Chou et al. show that palbociclib significantly induces apoptosis and also enhances the apoptotic effect of gemcitabine but only in a subset of pancreatic tumor models with high RB expression [92].

Combination treatment with oxaliplatin and the CDK4/6 inhibitor palbociclib synergistically inhibited CCA cell proliferation and prevented the emergence of CDK4/6i and oxaliplatin-resistant CCA. This drug combination also exerted suppressive and apoptotic effects on CCA in the in vitro 3-D cultures, patient-derived organoids, and in vivo xenograft CCA models [111]. 

A recent study explored the combination of CDK4 inhibitors and radiotherapy on CCA cells and reported that palbociclib affected the kinetics of DNA repair and enhanced the radiation sensitivity of hepatocellular carcinoma and CCA cells. Importantly, they found that palbociclib inhibits ataxia telangiectasia-mutated kinase, the key upstream kinase responding to radiotherapy-induced double-strand breaks [112].

An important recent report offers some resolution to these conflicting results by showing that the timing of administration is of crucial importance for the combination effect: the sequential administration of CDK4/6 inhibitors after, but not before, taxanes cooperated to prevent cellular proliferation in PDAC cells, PDTXs, and genetically engineered mice with *KRAS* and *CDKN2A* mutations [113]. Furthermore, CDK4/6 inhibitor post-treatment has been shown to enhance the action of several other classes of chemotherapeutic compounds, such as gemcitabine, 5-fluorouracil, cisplatin, etoposide, irinotecan monastrol, and Bl2536 [113]. This effect is mediated by the involvement of the CDK4/6–RB axis in DNA repair and recovery from mitotic stress [114]. These results strongly indicate that CDK4/6is hold huge potential to be applicable in the clinic as an addition to chemotherapy or radiotherapy or as maintenance therapy to be continued when chemotherapy has to stop due to cumulative toxicity.

### 2.5. CDK4/6is Impact on Immunogenic Mechanisms

Drug combinations can not only overcome drug resistance but also increase the clinical indications of the CDK4/6 inhibitor. Senescent cells acquire a pro-inflammatory senescence-associated secretory phenotype (SASP) that leads to their elimination mediated by macrophages and CD4+ T-cells [115]. This immune response is also elicited by oncogene-induced senescence [116]. Recent studies suggested that CDK4/6is may also promote the cytotoxic T-cell-mediated clearance of tumor cells and a tumor-suppressive immune microenvironment [117]. CDK4/6is were also shown to increase tumor immunogenicity by several mechanisms, increasing the tumor infiltration by effector T-cells, boosting the activation of these T-cells, and reducing the proliferation of regulatory T-cells [105,118]. The treatment of PDAC PDTX models with MEK and CDK4/6 inhibitors was demonstrated to have a profound impact on the myeloid and T-cell populations within the tumor compartment, eliciting sensitivity to anti-PD1 (programmed cell death protein 1) therapy in immune-competent models [119]. Another study using mouse models has shown that a combination of MEK and CDK4/6 inhibitors induces RB-mediated senescence to produce a SASP that includes pro-angiogenic factors that promote tumor vascularization, which in turn enhances drug delivery and the efficacy of cytotoxic gemcitabine chemotherapy. In addition, SASP-mediated endothelial cell activation stimulates the accumulation of CD8+ T-cells into otherwise immunologically ‘cold’ tumors, sensitizing tumors to PD-1 checkpoint blockade [120].

These results suggest that CDK4/6-induced senescence in pancreatic cancer can establish emergent susceptibilities to otherwise ineffective chemo- and immunotherapies through SASP-dependent effects on the tumor vasculature and immune system [120].

## 3. Challenges in the Clinical Use of CDK4/6is in Pancreatobiliary Cancers

The list of clinical studies investigating the effects of CDK4/6 inhibitors on CCA and PDAC is limited so far to three active and three completed studies presented in Table 1. The study results are available for two studies. A phase 1b study (NCT02501902) that tested the combination of palbociclib and nab-paclitaxel in patients with metastatic PDAC showed that although the combination regimen was tolerated in metastatic PDAC patients, it did not meet the pre-specified efficacy threshold [121].

Another phase 1b study that tested the Notch inhibitor in combination with other anticancer drugs in patients with advanced or metastatic solid tumors (NCT02784795) revealed that the Notch inhibitor combined with abemaciclib was poorly tolerated, leading to lowered dosing and disappointing clinical activity in patients with advanced or metastatic solid tumors, including CCA [122].

A major impediment to the effective treatment of patients with pancreatobiliary cancer is their extensive genetic heterogeneity and genomic plasticity that allows them to evade most therapeutic agents by bypassing some signaling pathways and thus becoming resistant [123]. Moreover, their stroma exhibits a strong desmoplastic feature, preventing drug access to tumoral cells [124,125]. Palbociclib has been shown to modulate extracellular matrix organization in vitro and in vivo, and the effects have been associated with decreased expression of α-SMA (alpha-smooth muscle actin), a marker of activated pancreatic stellate cells and stromal activation [92]. These effects are independent of the antiproliferative activity of palbociclib. Whether the observed changes in extracellular matrix mechanics induced by CDK4/6is may influence therapeutic sensitivity remains unknown and warrants further investigation. In addition, angiogenesis has been shown to be a CDK4/6-dependent process and combined CDK4/6i and MEKi therapy-induced SASP could increase the delivery of chemotherapy by inducing vascular remodeling [120].

Thus, before further testing the effects of CDK4/6is on pancreatobiliary cancers in clinical trials, it is crucial to better understand how CDK4/6is affect the tumoral stroma and tumoral vascularization.

The disappointing results of the initial clinical studies underscore the need for biomarker-driven trials to discover the subgroups of patients that would benefit the most from CDK4/6i therapies. Furthermore, as CDK4/6i monotherapy showed limited efficiency and the combination therapies hold promise to counteract CDK4/6i resistance, the metronomic regimen emerges as a critical and significant contributor to the success of combination therapy and should be considered in future clinical trial designs.

Finally, the resistance to CDK4/6 inhibitors could be overcome with the use of PROTACs (proteolysis targeting chimeras) technology. This innovative approach enables the selective degradation of targeted kinases, combining a ligand for the targeted protein and an E3 ligase-recruiting ligand, that are connected by a linker [126]. PROTAC technology aims to induce the proteasomal degradation of the target, instead of inhibiting it. The composition of PROTACs determines degradation selectivity and influences the stability and conformation of the ternary complex. Even though the single inhibitor acts to a comparable extent on the two kinases, several studies achieved the successful selective degradation of CDK4 or CDK6, depending on the type of CDK4/6 inhibitor and linker used [127,128,129]. CDK4/6 PROTACs with their high potency, relatively low toxicity, and no resistance appear to be a promising anticancer strategy to be tested in pancreatobiliary cancers.

## 4. Potential Biomarkers for Personalizing CDK4/6 Target Therapy

Several biomarkers, such as RB protein, CDKN2A, Cyclin D1 (CCND1), Cyclin E1 (CCNE1), high CDK4/6 expression, and the CDK4 phosphorylation status have emerged as being associated with CDK4/6 therapy from the current literature.

### 4.1. RB Protein

The activity of CDK4/6 requires a functional RB protein, and consequently, tumors that do not express functional RB should be resistant to these drugs. Loss of RB function has been proven to be a primary cause of CDK4/6i intrinsic and acquired resistance. Based on this, an intact RB and high RB expression appeared as one of the first candidate biomarkers to predict CDK4/6i responsiveness in preclinical cancer models [89,92,93,130]. However, the biomarker analyses in randomized PALOMA-2 and PALOMA-3 clinical trials in breast cancer failed to show a statistically significant correlation between RB and a benefit from CDK4/6 inhibitors [46]. Researchers speculated that acquired resistance to CDK4/6 inhibition might occur as a consequence of RB mutations, but not the loss of RB. It should be noted that the design of the study excluded triple-negative breast tumors in which those defects are more frequent.

### 4.2. Cyclin D1

Studies have demonstrated that amplification of the cyclin D1 gene could make some cancers more dependent on the CDK4/6 pathway and more vulnerable to CDK4/6 inhibition. However, in models of PDAC and CCA, elevated cyclin D1 expression can contribute to resistance [89,95,99]. In both ER-positive and HER2-positive breast cancers, cyclin D1 overexpression was observed. However, data from clinical trials did not show any direct correlations between the level of cyclin D1 and responsiveness to palbociclib [131].

### 4.3. Cyclin E

Cyclin E expression is induced by activated E2F1 and forms complexes with CDK2 that further phosphorylate RB, leading to a positive feedback loop favoring progression in the cell cycle. Thereby, as increasing the CDK2 activity bypasses the need for CDK4 activation, alterations leading to this activation can be the source of CDK4/6 inhibitor resistance. Indeed, high levels of *CCNE1* gene expression (often linked to *CCNE1* gene amplification) were correlated with resistance to palbociclib in the clinical setting [132]. Cyclin E has been shown to be elevated in PDAC models that are starting to progress on treatment [95]. This suggests that the co-targeting of CDK2 and CDK4/6 could be an alternative strategy to overcome resistance to CDK4/6is, which could be tested in the future. This strategy was shown to be promising as a combination of CDK4/6is with indisulam—a sulfonamide with anticancer effects—led to synergistic senescence induction and sensitized various cell lines to senolytic drugs [133].

### 4.4. Phosphorylation Status of CDK4

In breast and mesothelioma cancer cell lines, the phosphorylation status of Thr172 of CDK4 has been shown to correlate with sensitivity to palbociclib [91,133]. To alleviate the difficulty of the detection of labile phosphorylation events in formalin-fixed, paraffin-embedded (FFPE) tissue samples associated with the low cellular concentration of Thr172-phosphorylated CDK4 [24], Raspé et al. identified a gene expression signature of 11 genes that correlated with the Thr172 phosphorylation status and correctly predicted the CDK4 modification profile to palbociclib in 49 out of 52 breast tumors analyzed and sensitivity in 20 of 25 cell lines tested [91]. Furthermore, discordances between sensitivity observations and prediction were due to rare combinations of specific molecular defects, such as loss of the CDKN2A locus combined with the amplification of CCNE1. Importantly, both CCNE1 and CDKN2A are key components of the prediction signature.

### 4.5. CDKN2A

**Figure 7:**
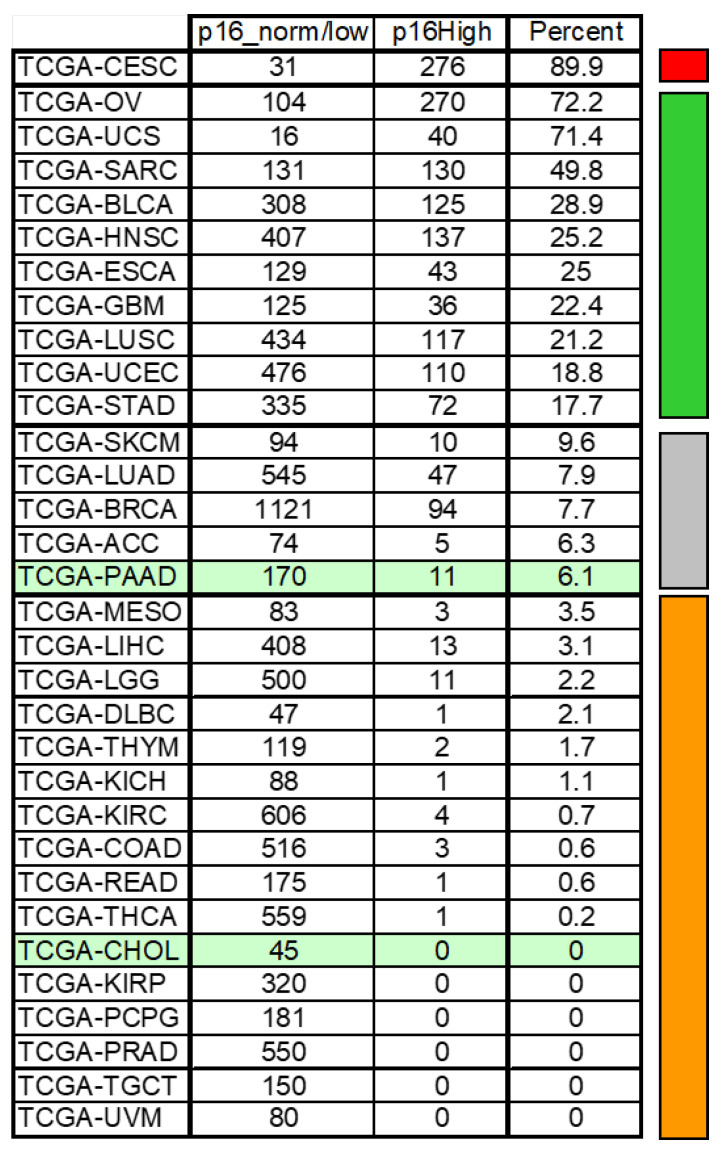
Proportion of tumors with an elevated CDKN2A level (TCGA database). The tumor types analyzed in the TCGA study are indicated in the first column. The numbers of tumors of each study with a low or normal CDKN2A expression is indicated in the second column. The numbers of tumors with elevated CDKN2A expression (comparable to the expression reached in HPV+ tumors) are indicated in the third column. The proportion with respect to the total of tumors with elevated CDKN2A is indicated in the forth column ("Percent") for each study. Tumors of the pancreas or cholangiocarcinoma are highlighted in green. The color code on the right indicates whether a predictive test would be needed to select responsive patients. In red, a test is unlikely to be useful because almost 90% of the tumors have elevated CDKN2A and likely lacks phosphorylated CDK4. CDK4 inhibitors should not be considered to treat patients with this type of tumor. In green, a test is absolutely required to identify potentially responsive patients. In grey, a test may be required to identify unresponsive patients after an eventual stratification by molecular subtypes. In orange, a test is probably not useful as less than 5% of the tumors have elevated CDKN2A likely associated with absent phosphorylation of CDK4. The use of CDK4 inhibitors to treat these tumors is worthwhile to consider as most of them will be sensitive to the drug, at least initially.

High expression of CDKN2A and the accumulation of p16 was consistently observed in resistant breast cancer cell lines [91,134]. This was confirmed in other tumor types, such as mesothelioma [135], undifferentiated thyroid cancer, and head and neck squamous carcinomas (unpublished data). CDKN2A status is particularly informative as, probably through an indirect ill-defined mechanism, increased E2F activity enhances the expression of the mRNA encoding the p16 protein [136,137,138,139]. Accordingly, the proportion of tumors of the TCGA study with an elevated CDKN2A level can be a good indicator of the proportion of tumors intrinsically resistant to CDK4is. As shown in Figure 7, this proportion is variable. It is highest in cervix cancer mostly caused by human papilloma virus (HPV) infection leading to RB function disruption and high CDKN2A expression in about 90% of the tumors. By contrast, no CCAs with high CDKN2A expression were reported in the TCGA. At these extremes of the spectrum, stratification of the tumors by performing a diagnostic test has little relevance. The proportion of tumors with elevated CDKN2A expression is higher in breast cancer (8%) and pancreatic cancer (6.1%), but remains low. A diagnostic test may nevertheless be required in particular in the triple negative breast cancers that are excluded from the indication of the drug because of the high proportion of intrinsically resistant tumors due to frequent RB defects or *CCNE1* amplification among this subtype. A diagnostic test will be unavoidable before treating sarcomas or bladder cancers for example.

*CDKN2A* loss or mutation found in a wide array of malignancies is a frequent event in PDAC and CCA and may lead to increased CDK activity. Palbociclib showed promising activity in patients with *CDKN2A*-mutated non-small-cell lung cancer [140]. However, palbociclib monotherapy tested in a small number of patients with advanced pancreatic or biliary cancers, preselected for *CDKN2A* loss or mutation, did not show any clinical activity [141].

### 4.6. CDK6 and CDK4 Gene Amplification

The acquired amplification of the *CDK6* gene has been associated with resistance to abemaciclib in breast cancer cell lines [128,129,142]. Interestingly, the overexpression of CDK4 was not observed in the same study and the enforced overexpression of CDK4 did not promote inhibitor resistance [142]. The impact of CDK4 silencing on sensitivity to palbociclib was also more recently studied in ER+ breast cancer and PDAC models [143]. They showed that the depletion of CDK4 by RNAi cooperates with palbociclib to reduce proliferation. Conversely, the overexpression of cyclin D1 and CDK4 decreases sensitivity to palbociclib.

## 5. Conclusions

The complex genetic heterogeneity of CCA and PDAC contribute to the ability of these cancers to circumvent current therapies and become resistant. This underscores the need for a more stratified therapeutic approach instead of a one-size-fits-all therapeutic treatment strategy. Before translating the preclinical findings on the utility of CDK4/6is for CCA and PDAC into clinical trials, it is important to decipher the molecular mechanisms of CDK4/6is action and their effects on cell cycle arrest in a cancer-specific context, taking into account the effects of CDK4/6 inhibition on the tumoral microenvironment and angiogenesis. Identifying biomarkers in patients reflecting resistance or the response to CDK4/6 inhibition would open the door for the development of new combination therapies in clinical trials.

## Figures and Tables

**Table 1:** Clinical trials evaluating the role CDK4/6is in pancreatobiliary cancers. Number of subjects (N).

NCT Number	Title	Status	Interventions	Conditions	N
NCT03454035	Ulixertinib/Palbociclib in Patients with Advanced Pancreatic and Other Solid Tumours	Active, not recruiting	Drug: Ulixertinib Drug: Palbociclib	Tumor, Solid Pancreatic Cancer Melanoma	45
NCT03339843	Multiorgan Metabolic Imaging Response Assessment of Abemaciclib	Active, not recruiting	Drug: Abemaciclib	Esophageal Adenocarcinoma Esophagus SCC Cholangiocarcinoma Urothelial/Bladder Cancer Endometrial Cancer	85
NCT03065062	Study of the CDK4/6 Inhibitor Palbociclib (PD-0332991) in Combination with the PI3K/mTOR Inhibitor Gedatolisib (PF-05212384) for Patients with Advanced Squamous Cell Lung, Pancreatic, Head & Neck and Other Solid Tumours	Recruiting	Drug: Palbociclib Drug: Gedatolisib	Lung Cancer Squamous Cell Solid Tumors Head & Neck Cancer Pancreatic Cancer	96
NCT02981342	A Study of Abemaciclib (LY2835219) Alone or in Combination with Other Agents in Participants with Previously Treated Pancreatic Ductal Adenocarcinoma	Completed	Drug: Abemaciclib Drug: LY3023414 Drug: Gemcitabine Drug: Capecitabine	Lung Cancer Squamous Cell Solid Tumors Head and Neck Cancer Pancreatic Cancer	106
NCT02784795	Study of LY3039478 in Participants with Advanced or Metastatic Solid Tumours	Completed	Drug: LY3039478 Drug: Taladegib Drug: Abemaciclib Drug: Cisplatin Drug: Gemcitabine Drug: Carboplatin Drug: LY3023414	Solid Tumor Breast Cancer Colon Cancer Cholangiocarcinoma Soft Tissue Sarcoma	94
NCT02501902	Dose-Escalation Study of Palbociclib + Nab-Paclitaxel In mPDAC	Completed	Drug: Palbociclib Drug: Nab-Paclitaxel	Metastatic Pancreatic Ductal Adenocarcinoma	76

## Glossary

AKT	protein kinase B
AP1	activator protein 1 (transcription factor)
BrdU	5-bromo-2-deoxyuridine
CCA	cholangiocarcinoma
CCND	cyclin D
CCNE1	cyclin E1
CDK2	cyclin dependent kinase 2
CDKN2A/B	cyclin-dependent kinase inhibitor 2A/B gene
CDK4/6	cyclin-dependent kinase 4/6
CDK4/6i	cyclin-dependent kinase 4/6 inhibitor
CDK7	cyclin dependent kinase 7
eCAA	extrahepatic cholangiocarcinoma
ECM	extracellular matrix
EdU	5-ethynyl-2′-deoxyuridine
E2F	E2F transcription factor
FACS	Fluorescence-activated Cell Sorting
FAK	focal adhesion kinase
FFPE	formalin-fixed, paraffin-embedded
FOLFOX	chemotherapeutic regimen composed of folinic acid, fluorouracil, and oxaliplatin
FOLFIRINOX	chemotherapeutic regimen composed of irinotecan, oxaliplatin and leucovirin-modulated fluorouracil
HPV	Human papillomavirus
HuR	human antigen R
iCC	intrahepatic cholangiocarcinoma
IGF1	insulin-like growth factor 1
KRAS	kirsten rat sarcoma virus
mTOR	mammalian target of rapamycin
MEK	mitogen-activated protein kinase
MEKi	mitogen-activated protein kinase inhibitor
PDAC	pancreatic ductal adenocarcinoma
PDTX	patient-derived tumor xenographt
PD-1	programmed cell death protein 1
PI3K	phosphatidylinositol 3-kinase
p21	cyclin-dependent kinase (Cdk) inhibitor p21
p27	cyclin-dependent kinase (Cdk) inhibitor p27
PROTAC	proteolysis targeting chimera
RB	retinoblastoma protein
SASP	senescence-associated secretory phenotype
SMAD	suppressor of mothers against decapentaplegic
α-SMA	alpha-smooth muscle actin
TCGA	The Cancer Genome Atlas Program
TGF-β	transforming growth factor β
TβRI	type-I transforming growth factor-β receptor
YAP1	yes1-associated transcriptional regulator
